# Clinical value of probiotics as adjunctive therapy for diabetic nephropathy: a meta-analysis and trial sequential analysis

**DOI:** 10.3389/fnut.2026.1863111

**Published:** 2026-09-17

**Authors:** Xiangning Huang, Xinyu Yang, Yunfeng Yu, Jiawang Huang, Wang Tao, Rong Yu, Xiu Liu

**Affiliations:** 1 School of Traditional Chinese Medicine, Hunan University of Chinese Medicine, Changsha, China; 2 Department of Endocrine, The First Hospital of Hunan University of Chinese Medicine, Changsha, China

**Keywords:** clinical efficacy, diabetic nephropathy, meta-analysis, probiotics, systematic review

## Abstract

**Objective:**

To comprehensively assess the clinical effectiveness of probiotic supplementation in individuals with diabetic nephropathy (DN).

**Methods:**

Randomized controlled trials (RCTs) evaluating probiotics for DN were retrieved from eight databases from inception to January 2026. Data on study characteristics, outcome measures, and risk of bias were independently extracted. Meta-analyses were conducted using Review Manager version 5.3, while trial sequential analysis (TSA) was performed with TSA version 0.9.5.10 beta. Potential publication bias was examined using Egger’s regression test.

**Results:**

Nine RCTs encompassing 644 participants met the inclusion criteria. Pooled analyses indicated that probiotic supplementation was associated with significant reductions in serum creatinine (mean difference [MD] −0.10 mg/dL, 95% confidence interval [CI] −0.14 to −0.06), blood urea nitrogen (BUN) (MD − 2.76 mg/dL, 95% CI − 5.40 to −0.11), fasting plasma glucose (MD − 0.63 mmol/L, 95% CI − 0.80 to −0.46), 2-h postprandial blood glucose (2 h PBG) (MD − 0.66% mmol/L, 95% CI −1.10 to −0.22), glycated hemoglobin (HbA1c) (MD −0.40%, 95% CI −0.72 to −0.09), triglycerides (MD −19.17 mg/dL, 95% CI −35.14 to −3.20), total cholesterol (MD −11.68 mg/dL, 95% CI −20.37 to −2.99), low-density lipoprotein cholesterol (MD −12.72 mg/dL, 95% CI −18.76 to −6.67), high-sensitivity C-reactive protein (MD −1.69 mg/L, 95% CI −2.38 to −1.00), and malondialdehyde (MD − 0.52 μmol/L, 95% CI −0.91 to −0.13). However, no statistically significant improvements were detected in estimated glomerular filtration rate, 24-h urinary protein excretion, insulin levels, high-density lipoprotein cholesterol, or total antioxidant capacity. TSA supported the reliability of all significant findings except for BUN and triglycerides. Egger’s test revealed no evidence of publication bias for most outcomes.

**Conclusion:**

Probiotics reduce circulating inflammation and oxidative stress levels in patients with DN, highlighting their potential as an adjunctive therapy, although their effects on glycolipid metabolism and kidney function appear to be modest. Nevertheless, given the limitations of small sample sizes and regional concentration, these findings warrant further validation through multicenter RCTs involving diverse ethnic populations.

**Systematic review registration:**

The systematic review was prospectively registered in PROSPERO (CRD420261375372). The registration is publicly accessible at https://www.crd.york.ac.uk/prospero/.

## 1 Introduction

Diabetic nephropathy (DN) is one of the most common microvascular complications of diabetes mellitus (DM), with a prevalence of up to 30–40% among patients with diabetes (1). It has been reported that the cardiovascular mortality of patients with DN is increased by approximately 60% compared with those with DM without kidney involvement (2), and up to 30–50% of patients with DN eventually progress to end-stage renal disease (ESRD) (3). According to the Global Burden of Disease study, the burden of DN has risen sharply from 1990 to 2021, with global deaths and disability-adjusted life years attributable to DN increasing by 189.6 and 138.6%, respectively (4). The pathogenesis of DN is closely associated with mechanisms such as glomerular hyperfiltration, activation of the renin–angiotensin–aldosterone system, tubulointerstitial inflammation and fibrosis, and podocyte dysfunction (5, 6). Current guidelines recommend that first-line treatment for DN primarily focuses on glycemic control, blood pressure reduction, and proteinuria management. Commonly used medications include sodium–glucose cotransporter-2 inhibitors, angiotensin-converting enzyme inhibitors or angiotensin II receptor blockers, and mineralocorticoid receptor antagonists (7, 8). With the development of high-throughput sequencing technologies (9), increasing attention has been paid to the role of the gut microbiota in DN, particularly following the emergence of the “gut–kidney axis” concept (10–12).

Clinical evidence indicates that the composition and relative abundance of gut microbiota differ significantly among patients with DN, patients with DM without kidney damage, and healthy individuals. At the genus level, compared with age- and sex-matched healthy controls and patients with DM, patients with DN exhibit decreased abundance of *Gemmiger* and increased abundance of *Flavonifractor* and *Eisenbergiella* (13). *Flavonifractor* and *Eisenbergiella* have been shown to promote the production of pro-inflammatory cytokines such as IL-1β, IL-6, and TNF-α, thereby contributing to systemic inflammation (14, 15). Subsequent clinical studies have demonstrated that, compared with healthy controls, patients with DM, and those with chronic kidney disease, patients with DN show a significantly increased abundance of *Escherichia coli*, which is positively correlated with blood glucose and urinary protein levels (11). Further *in vivo* studies have confirmed that gut microbiota may influence the onset and progression of DN through bacterial translocation, extracellular vesicles, and microbial metabolites (12, 16, 17). For example, in a DN mouse model, gut-derived bacteria such as *Klebsiella oxytoca* translocate into the bloodstream and reach the kidneys, where they activate kidney injury molecule-1 (12). In addition, oral administration of fecal-derived extracellular vesicles from diabetic rats for 16 weeks activates Caspase-11 in the kidneys of C57BL/6 J mice, leading to tubulointerstitial inflammation and renal injury (17). Moreover, gut microbiota-derived metabolites, including indoxyl sulfate, trimethylamine N-oxide, and p-cresyl sulfate, promote renal dysfunction by affecting glucose homeostasis, inflammation, RAAS activation, and immune responses (16). Conversely, probiotics may restore gut microbiota dysbiosis in patients with DN, thereby reducing circulating uremic toxins such as p-cresyl sulfate, IAA-O-glucuronide, and 1-methylinosine (18). These findings suggest that probiotics may improve the prognosis of DN through modulation of the gut–kidney axis.

Recent meta-analyses have suggested that probiotics may improve glycolipid metabolism and renal function in patients with DN, providing preliminary evidence for their adjunctive therapeutic role (19–26). However, these studies have reported inconsistent findings across several outcomes. Regarding glycemic metabolism, Liu et al. (19), Tarrahi et al. (20) and AbdelQadir et al. (23) reported significant improvements in fasting blood glucose (FBG) and insulin-related parameters following probiotic supplementation, whereas Dai et al. (22) failed to demonstrate statistically significant effects. Similarly, findings regarding lipid metabolism remain controversial. Liu et al. (19), Moravejolahkami et al. (24) and Zheng et al. (25) observed significant reductions in triglycerides (TG), total cholesterol (TC) and low-density lipoprotein cholesterol (LDL-C), while AbdelQadir et al. (23) and Dai et al. (22) found no significant improvements in these lipid parameters. Regarding renal outcomes, previous meta-analyses have also yielded inconsistent findings. Tarrahi et al. (20) reported a significant reduction in serum creatinine (SCR) following probiotic supplementation, whereas Liu et al. (19) observed significant improvements in both SCR and blood urea nitrogen (BUN). Wang et al. (26) further demonstrated that probiotics significantly improved SCR, estimated glomerular filtration rate (eGFR), and BUN. In contrast, AbdelQadir et al. (23) failed to identify significant effects of probiotics on major renal function indicators. These conflicting results raise concerns regarding the robustness of existing evidence.

More importantly, previous meta-analyses have several inherent limitations. Specifically, Tarrahi et al. (20), Bohlouli et al. (21), Dai et al. (22), AbdelQadir et al. (23) and Wang et al. (26) included DN complicated by ESRD, which may introduce dialysis-related confounding factors. Liu et al. (19) excluded patients with a disease duration longer than 10 years, potentially limiting the generalizability of their findings. Bohlouli et al. (21) and Moravejolahkami et al. (24) included trials exclusively conducted in Iran, which may limit the generalizability of their findings to populations with different ethnic backgrounds. Zheng et al. (25) evaluated the effects of probiotics, prebiotics, and synbiotics among patients with chronic kidney disease, including heterogeneous CKD populations rather than DN-specific patients, making it difficult to determine the independent therapeutic efficacy of probiotics in diabetic nephropathy. Furthermore, none of these meta-analyses applied trial sequential analysis (TSA) to adjust for random errors, thereby increasing the risk of bias and false-positive results. Given these methodological limitations and inconsistent findings, a more rigorous and comprehensive evidence synthesis is urgently needed to clarify the true efficacy of probiotics in DN.

Therefore, the present study aimed to provide a more precise and robust meta-analysis by applying stricter inclusion criteria to minimize confounding factors and reduce potential literature omission, as well as incorporating TSA to control for random errors. This approach is expected to help resolve existing inconsistencies and offer a more comprehensive perspective on the clinical application of probiotics in DN.

## 2 Methods

This study strictly followed the Preferred Reporting Items for Systematic reviews and Meta-Analyses 2020 (PRISMA 2020) guidelines (27). The study protocol was prospectively registered in the PROSPERO database (registration number: CRD420261375372). The registration is publicly accessible at https://www.crd.york.ac.uk/prospero/.

### 2.1 Inclusion and exclusion criteria

Studies were considered eligible if they satisfied all of the following conditions: (i) Participants: adults aged 18 years or older with a confirmed diagnosis of DN based on established clinical and/or laboratory criteria; (ii) Intervention: administration of probiotics in combination with standard therapy; (iii) Comparator: standard therapy alone without probiotic supplementation; (iv) Outcomes: reporting at least one of the following outcome measures: renal function parameters (SCR, 24-h Urine Protein [24 h UP], eGFR, BUN); glycemic indices (FBG, 2-h postprandial blood glucose [2 h PBG], glycated hemoglobin [HbA1c], insulin); lipid profiles (TG, TC, LDL-C, high-density lipoprotein cholesterol [HDL-C]); and inflammatory or oxidative stress markers (high-sensitivity C-reactive protein [hs-CRP], total antioxidant capacity [TAC], malondialdehyde [MDA]); (v) Study design: randomized controlled trials (RCTs).

Studies were excluded if they met any of the following criteria: (i) duplicate publications; (ii) insufficient or incomplete data that precluded extraction or quantitative synthesis; (iii) inclusion of participants diagnosed with uremia or ESRD.

### 2.2 Literature search strategy

A systematic and comprehensive literature search was conducted across PubMed, Embase, Web of Science, the Cochrane Library, China National Knowledge Infrastructure (CNKI), the China Science and Technology Journal Database (VIP), Wanfang Data, and SinoMed from database inception to January 1, 2026. The searches were conducted within the Topic or Title/Abstract fields, using the following search query: (Probiotic OR Probiotics OR Synbiotic OR Synbiotics OR Bifidobacterium OR Bifidobacteria OR Bacillus bifida OR Yeast OR *Saccharomyces cerevisiae* OR Saccharomyces italicus OR Saccharomyces oviformis OR S cerevisiae OR *S. cerevisiae* OR Saccharomyces uvarum var. melibiosus OR *Candida robusta* OR Saccharomyces capensis OR *Lactobacillus acidophilus* OR *Lactobacillus amylovorus* OR Lactobacill OR Lactic acid bacteria OR *Clostridium butyricum* OR Bacillus OR Natto Bacteria OR Streptococcus thermophiles OR Enterococcus) AND (Diabetic Nephropathy OR Diabetic Nephropathies OR Diabetic Kidney Disease OR Diabetic Kidney Diseases OR Diabetic Glomerulosclerosis OR Intracapillary Glomerulosclerosis OR Kimmelstiel Wilson Disease OR Nodular Glomerulosclerosis OR Kimmelstiel Wilson Syndrome). No restrictions were imposed on language or publication status. In addition, the reference lists of relevant reviews and all included articles were manually screened to identify potentially eligible studies not captured by the electronic search.

### 2.3 Study selection process

All retrieved records were imported into EndNote software for reference management and removal of duplicates. Two reviewers independently screened titles and abstracts to identify potentially relevant studies. Full-text articles were subsequently assessed for eligibility based on the predefined inclusion and exclusion criteria. Any discrepancies between reviewers were resolved through discussion, and when necessary, consultation with a third reviewer. The study selection procedure was summarized and presented using a PRISMA flow diagram.

### 2.4 Data extraction

Data extraction was independently performed by two reviewers using a standardized, predesigned extraction form. The following information was collected: (i) study characteristics: first author, year of publication, country, and study design; (ii) participant characteristics: sample size, age, sex distribution, body weight, and duration of DN; (iii) intervention details: probiotic strains, dosage, administration frequency, and intervention duration; (iv) methodological information: funding sources and reported conflicts of interest. Any disagreements during data extraction were resolved through consensus or adjudication by a third reviewer.

### 2.5 Risk of bias assessment

The methodological quality of the included RCTs was independently assessed by two reviewers using the Cochrane Collaboration’s Risk of Bias tool (RoB 1.0). Seven domains were evaluated: random sequence generation, allocation concealment, blinding of participants and personnel, blinding of outcome assessment, completeness of outcome data, selective reporting, and other potential sources of bias. Each domain was rated as having a low, high, or unclear risk of bias. Discrepancies in assessments were resolved by discussion or, if required, by consultation with a third reviewer.

### 2.6 Statistical analysis

All statistical analyses were performed using Review Manager software (version 5.3) and Stata (version 17.0). For continuous variables, pooled effects were expressed as mean differences (MDs; equivalent to weighted mean differences [WMDs] in conventional meta-analysis terminology) or standardized mean differences (SMDs), each accompanied by 95% confidence intervals (CIs). Dichotomous outcomes were summarized using risk ratios (RRs) with corresponding 95% CIs. Between-study heterogeneity was quantified using the *I*^2^ statistic. An *I*^2^ value greater than 50% was considered indicative of substantial heterogeneity, prompting the use of a random-effects model; otherwise, a fixed-effects model was applied. A two-sided *p*-value of less than 0.05 was regarded as statistically significant.

Sensitivity analyses were performed using a leave-one-out approach to evaluate the robustness of the pooled estimates across all outcomes. For outcomes showing substantial heterogeneity (*I*^2^ > 50%), this approach was additionally used to explore potential sources of heterogeneity by identifying studies that contributed disproportionately to between-study variability. The robustness of the pooled effects was assessed by examining whether exclusion of individual studies materially altered the magnitude, direction, or statistical significance of the overall estimates.

In addition, for outcomes exhibiting considerable heterogeneity and including five or more studies, prespecified subgroup analyses were undertaken according to clinically relevant characteristics, including sex, mean age, body weight, baseline HbA1c and serum creatinine levels, disease duration, type of probiotic preparation, and length of intervention. These analyses were designed to explore potential clinical contributors to heterogeneity and to further assess the consistency of the results across different subgroups.

To further control for random errors and assess the conclusiveness of cumulative evidence, TSA was performed using TSA software (version 0.9.5.10 beta). The required information size was calculated based on a prespecified anticipated effect size, a type I error rate of 5%, and a statistical power of 80%. Sequential monitoring boundaries were applied to determine whether the accumulated evidence was sufficient to draw definitive conclusions or whether additional trials were necessary. A result was considered conclusive if the cumulative Z-curve crossed the TSA monitoring boundary.

### 2.7 Publication bias

Potential publication bias was quantitatively assessed using funnel plots and Egger’s tests. Funnel plot asymmetry was evaluated qualitatively, while Egger’s test was used for quantitative assessment. A *p*-value below 0.05 in Egger’s test was considered indicative of statistically significant small-study effects. Because funnel plot asymmetry may also arise from factors other than publication bias, including heterogeneity and limited sample sizes, the results of funnel plots and Egger’s tests were interpreted together.

### 2.8 Certainty of evidence

The overall certainty of evidence for each outcome was evaluated using the Grading of Recommendations Assessment, Development and Evaluation (GRADE) framework. Evidence quality was categorized as high, moderate, low, or very low based on consideration of five domains: risk of bias, inconsistency, indirectness, imprecision, and publication bias.

## 3 Results

### 3.1 Study selection

The literature search yielded a total of 1,074 records from the electronic databases, including PubMed (*n* = 109), Embase (*n* = 158), the Cochrane Library (*n* = 119), Web of Science (*n* = 151), China National Knowledge Infrastructure (CNKI; *n* = 158), VIP Database (*n* = 22), Wanfang Data (*n* = 265), and SinoMed (*n* = 92). During the review process, we excluded 375 duplicate records and 675 records that were unrelated to the research topic. The full texts of the remaining 24 articles were assessed for eligibility, resulting in the exclusion of 15 articles (two due to duplicated data and 13 for failure to meet the predefined intervention criteria). Ultimately, nine randomized controlled trials (RCTs) (28–36) were included in the quantitative synthesis. The detailed study selection process is illustrated in the PRISMA flow diagram (Figure 1).

**Figure 1:**
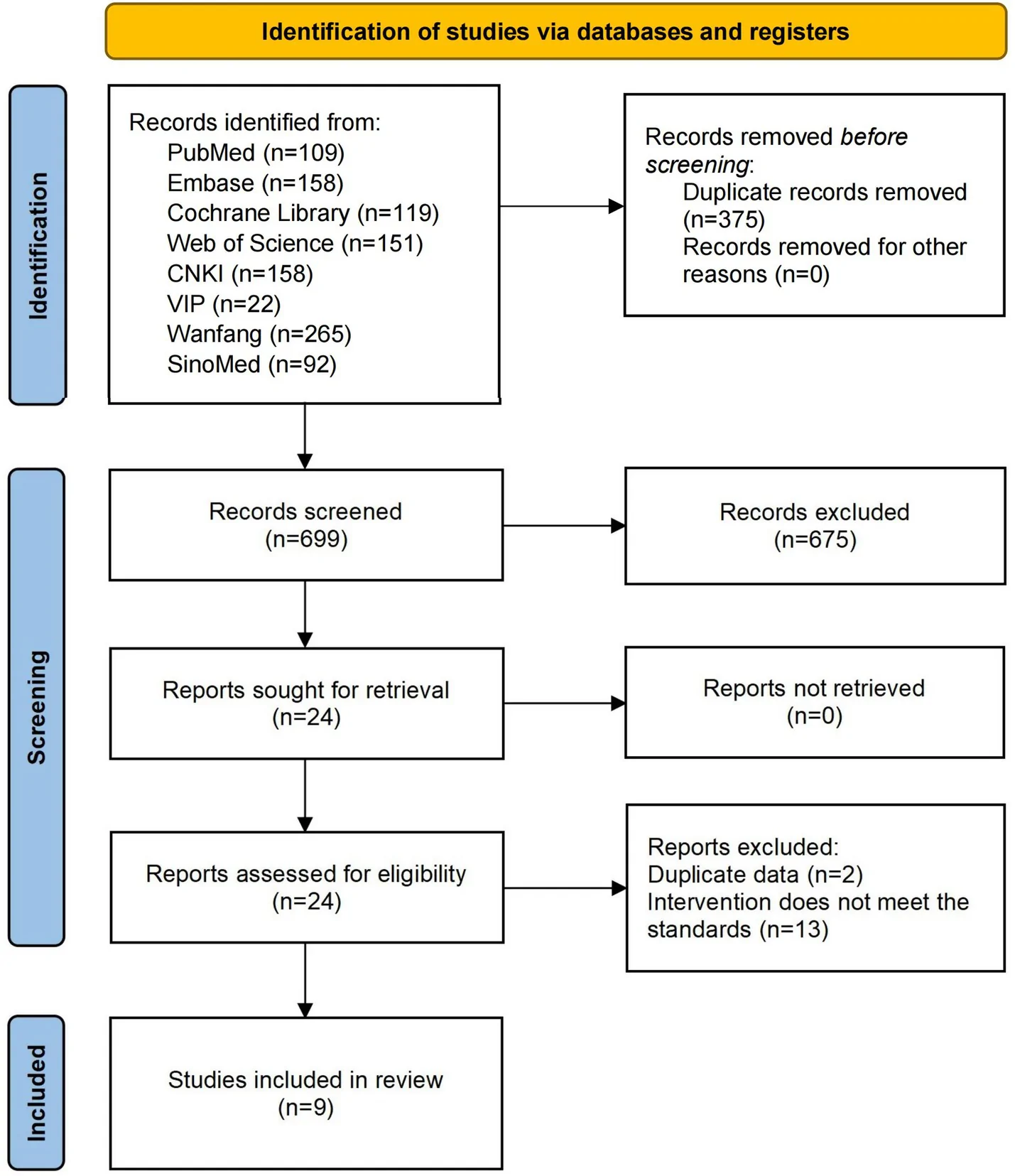
PRISMA flowchart. The flowchart outlines the stages of study identification, screening, eligibility assessment, and final inclusion for the meta-analysis. An initial search across eight electronic databases yielded a total of 1,074 records. After the removal of 375 duplicate records, 699 records were screened by title and abstract, resulting in the exclusion of 675 irrelevant records. The full texts of the remaining 24 reports were retrieved and assessed for eligibility. Among these, 15 reports were excluded because of duplicated data (*n* = 2) or interventions not meeting the predefined criteria (*n* = 13). Ultimately, 9 randomized controlled trials were included in the quantitative synthesis. PRISMA, Preferred Reporting Items for Systematic Reviews and Meta-Analyses; *n*, number of studies.

### 3.2 Basic characteristics of included studies

Nine RCTs comprising a total of 644 participants were included in this meta-analysis. Of these, 326 participants received probiotic supplementation in conjunction with standard therapy, while 318 participants were treated with standard therapy alone. Across the included studies, women accounted for an average of 50.43% of the study population. The mean age of participants was 58.69 years, and the average body weight was 73.35 kg. Baseline glycemic control, as indicated by HbA1c, ranged from 6.80 to 8.86%, while baseline serum creatinine levels varied between 0.82 and 3.27 mg/dL. The reported duration of DN ranged from 3.56 to 14.18 years. Regarding interventions, three trials employed single-strain probiotic preparations, whereas six trials used multi-strain formulations. The duration of probiotic administration varied from 4 to 24 weeks. The specific probiotic strains used across the included studies comprised Lactobacillus species (*L. plantarum* A7, *L. acidophilus*, *L. casei*, *L. lactis*, *L. bulgaricus*, *L. reuteri*, *L. fermentum*, and *L. infantis*), Bifidobacterium species (*B. bifidum*, *B. longum*, and *B. infantis*), as well as *Streptococcus thermophilus*, *Enterococcus faecalis*, *Bacillus coagulans*, and *Bacillus cereus*. The daily probiotic dosage ranged from 1.4 × 10^7^ CFU/day to 6 × 10^10^ CFU/day, with six trials administering ≥ 4 × 10^9^ CFU/day and three trials administering < 4 × 10^9^ CFU/day. The duration of probiotic administration varied from 4 to 24 weeks, with six trials lasting ≥ 12 weeks and three trials lasting < 12 weeks. Detailed characteristics of all included studies are summarized in Table 1.

**Table 1:** Basic characteristics of included studies.

Study ID	Country	Sample	Female (%)	Age (years)	Weight (kg)	HbA1c (%)	SCR (mg/dL)	Disease duration (years)	Intervention	Probiotics	Treatment duration (weeks)
Abbasi (2018) (28)	Iran	20	55.00	56.90	70.80	–	1.01	8.70	Probiotic soy milk 200 mL qd	4 × 10^9^ CFU/d *L. plantarum* A7	8
		20	45.00	53.60	71.60	–	1.03	6.90	Conventional soy milk 200 mL qd	–	8
Arani (2019) (29)	Iran	30	–	62.70	79.60	–	1.60	–	Probiotic honey 25 g qd	2.5 × 10^9^ CFU/d viable and heat-resistant probiotic *Bacillus coagulans* T4	12
		30	–	60.30	78.00	–	1.30	–	Honey 25 g qd	–	12
Firouzi (2015) (30)	Malaysia	68	45.59	52.90	74.60	–	0.82	–	Microbial cell preparation qd	6 × 10^10^ CFU/d (*L. acidophilus, L. casei, L. lactis, B. bifidum, B. longum* and *B. infantis*)	12
		68	50.00	54.20	76.60	–	0.85	–	Placebo qd	–	12
Guan (2022) (31)	China	35	45.71	60.63	–	8.86	1.46	3.56	Live combined bifidobacterium and lactobacillus tablets 2 g tid compound eosinophi-lactobacillus tablets 1 g tid	1 × 10^8^ CFU/d (*B. longum* 6 × 10^7^ CFU, *L. bulgaricus* 6 × 10^6^ CFU*, S. thermophilus* 6 × 10^6^ CFU and *L. acidophilus* 3 × 10^7^ CFU)	24
		35	42.86	61.52	–	8.78	1.48	3.61	None	–	24
Jiang (2021) (32)	China	42	64.29	55.96	–	7.32	–	–	Probiotic capsules qd	3.2 × 10^9^ CFU/d (*B. bifidum* 1.2 × 10^9^ CFU, *L. acidophilus* 4.2 × 10^9^ CFU and *S. thermophilus* 4.3 × 10^9^ CFU)	12
		34	64.71	56.12	–	7.92	–	–	Placebo qd	starch	12
Lu (2024) (33)	China	36	44.44	68.96	–	8.48	1.42	13.96	Live combined bifidobacterium, lactobacillus, enterococcus and *bacillus cereus* tablets 1.5 g tid	1.4 × 10^7^ CFU/d (*B. infantis* 4.5 × 10^6^ CFU, *L. acidophilus* 4.5 × 10^6^ CFU, *E. faecalis* 4.5 × 10^6^ CFU, *Bacillus cereus* 4.5 × 10^5^ CFU)	4
		36	50.00	69.57	–	8.55	1.40	14.18	None	–	4
Mafi (2018) (34)	Iran	30	–	58.90	69.30	6.80	1.30	–	Probiotic supplements qd	8 × 10^9^ CFU/d (*L. acidophilus* strain ZT-L1, *B. bifidum* strain ZT-B1, *L. reuteri* strain ZT-Lre, and *L. fermentum* strain ZT-L3)	12
		30	–	60.90	70.50	6.90	1.40	–	Placebo	–	12
Miraghajani (2017) (35)	Iran	20	–	56.90	70.84	–	–	8.70	Probiotic soy milk 200 ml qd	4 × 10^9^ CFU/d *L. plantarum* A7	8
		20	–	53.60	71.61	–	–	6.90	Conventional soy milk 200 ml qd	–	8
Tang (2020) (36)	China	45	51.11	55.82	–	6.92	2.98	9.28	Probiotic supplements bid	4.8 × 10^7^ CFU/d (*B. longum* 1 × 10^7^ CFU, *L. bulgaricus* 1 × 10^6^ CFU and *S. thermophilus* 1 × 10^6^ CFU)	12
		45	46.47	56.86	–	7.31	3.27	9.39	–	–	12

HbA1c, glycosylated hemoglobin; SCR, serum creatinine; CFU, colony-forming unit; *L., Lactobacillus*; *B., Bifidobacterium*; *S., Streptococcus*.

### 3.3 Risk of bias

The risk of bias assessment of the included trials, conducted using the Cochrane Collaboration’s RoB 1.0 tool, is summarized in Figure 2. All included studies reported appropriate methods for random sequence generation and were therefore judged to be at low risk of bias for this domain (28–36). Allocation concealment was rated as having an unclear risk of bias in four studies due to the absence of detailed information regarding concealment procedures (31, 33, 34, 36). Similarly, five studies did not report the implementation of blinding for participants and personnel (29, 31, 33, 35, 36), resulting in an unclear risk of bias for the domain of performance bias. Because all primary outcomes consisted of objective biochemical indicators, which are unlikely to be influenced by assessor subjectivity, blinding of outcome assessment was judged to be at low risk of bias across all studies (28–36). One study reported a dropout rate exceeding 20%, and was consequently assessed as having a high risk of bias related to incomplete outcome data (32). Selective reporting was evaluated as having an unclear risk of bias in four studies (30, 31, 33, 36), as their trial registration protocols were not publicly accessible. For other potential sources of bias, insufficient information was available to determine the presence or absence of additional bias; therefore, this domain was uniformly judged as unclear risk.

**Figure 2:**
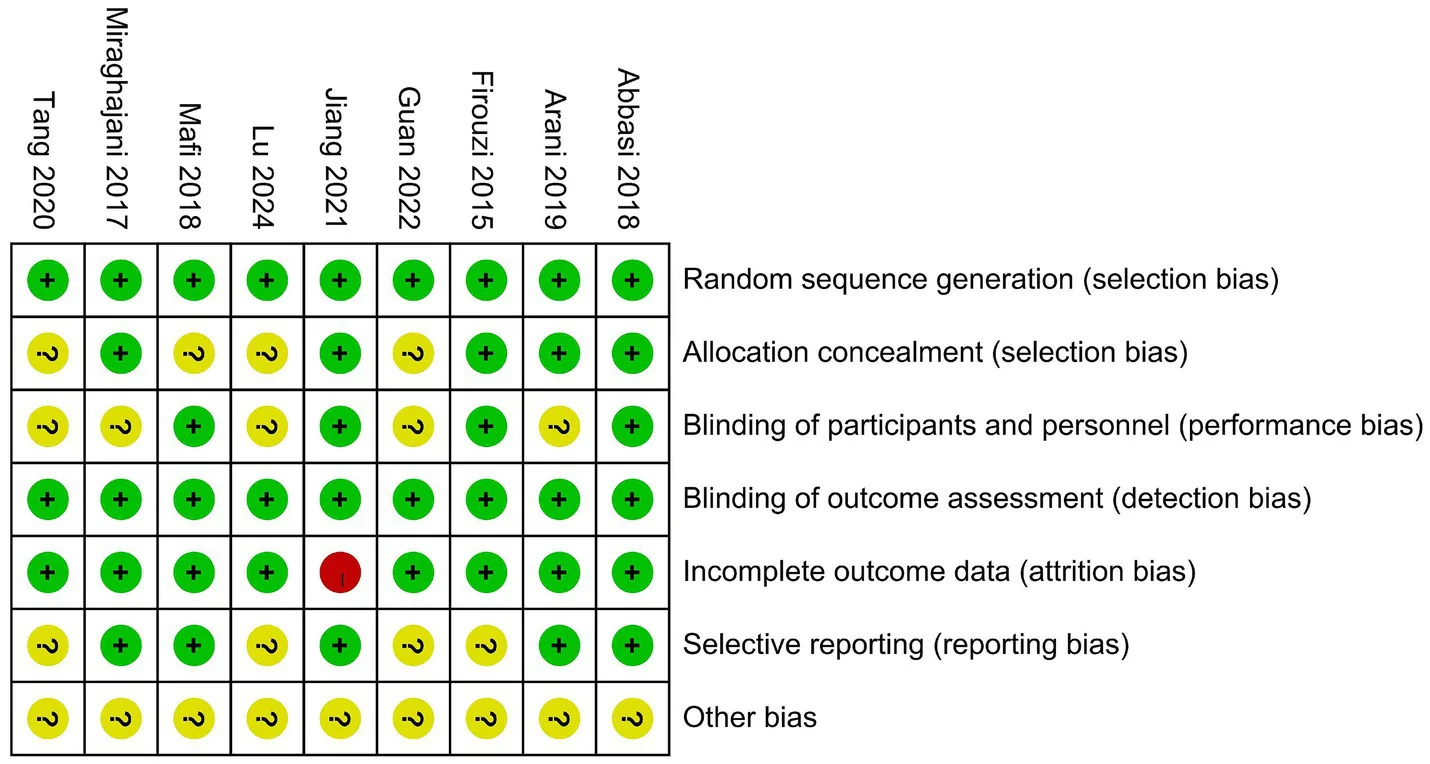
Risk assessment of bias. The assessment covers seven domains: random sequence generation, allocation concealment, blinding of participants and personnel, blinding of outcome assessment, completeness of outcome data, selective reporting, and other potential sources of bias. Green denotes low risk of bias, yellow denotes unclear risk of bias, and red denotes high risk of bias. Overall, the included studies were judged to have a moderate risk of bias, primarily due to insufficient reporting of allocation concealment in several trials. RCT, randomized controlled trial.

### 3.4 Meta-analysis

#### 3.4.1 Renal function outcomes

Compared with standard therapy alone, the probiotic supplementation was associated with a statistically significant reduction in SCR (MD −0.10 mg/dL, 95% CI −0.14 to −0.06, *p* < 0.00001, *I*^2^ = 36%) and BUN (MD −2.76 mg/dL, 95% CI −5.40 to −0.11, *p* = 0.04, *I*^2^ = 83%). However, no statistically significant differences were observed in 24 h UP (MD −0.24 g, 95% CI −0.48 to 0.01, *p* = 0.06, *I*^2^ = 41%) or eGFR (MD 6.06 mL/[min*1.73 m^2^], 95% CI −0.72 to 12.83, *p* = 0.08, *I*^2^ = 88%) between the probiotic and control groups (Figure 3).

**Figure 3:**
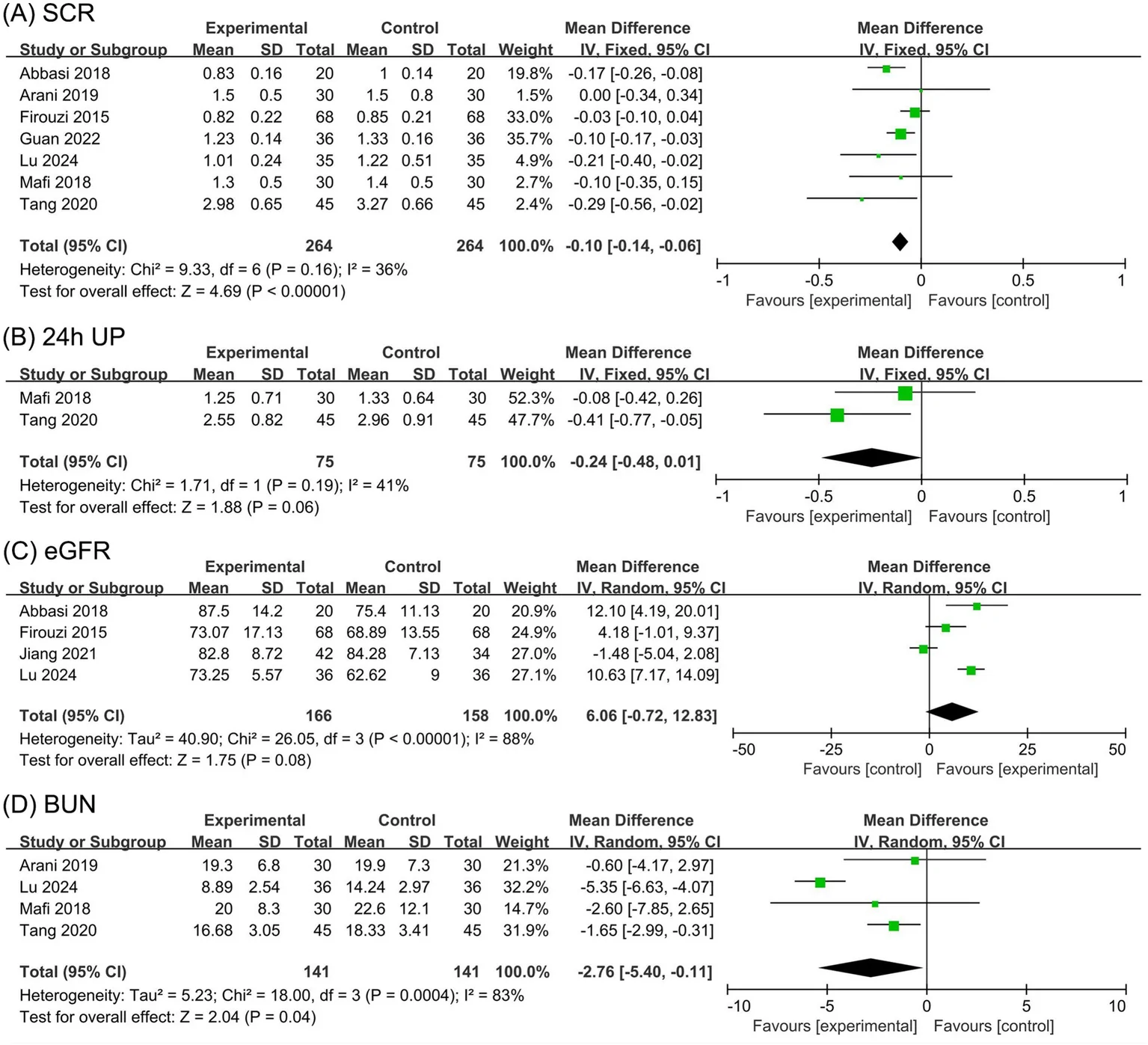
Forest plots of the meta-analysis on renal function outcomes: **(A)** SCR; **(B)** 24 h UP; **(C)** eGFR; **(D)** BUN. The diamond represents the pooled effect estimate; squares represent individual study estimates with size proportional to study weight. MD, mean difference; CI, confidence interval; SCR, serum creatinine; 24 h UP, 24-h urine protein; eGFR, estimated glomerular filtration rate; BUN, blood urea nitrogen.

#### 3.4.2 Glucose metabolism outcomes

Compared with standard therapy alone, probiotic supplementation was associated with significant reductions in glucose metabolism indicators, including FBG (MD −0.63 mmol/L, 95% CI −0.80 to −0.46, *p* < 0.00001, *I*^2^ = 0), 2 h PBG (MD −0.66 mmol/L, 95% CI −1.10 to −0.22, *p* = 0.004, *I*^2^ = 0), and HbA1c (MD −0.40%, 95% CI −0.72 to −0.09, *p* = 0.01, *I*^2^ = 82%). However, no statistically significant difference was observed in circulating insulin levels between the probiotic and control groups (MD −1.21 μIU/mL, 95% CI −3.32 to 0.90, *p* = 0.26, *I*^2^ = 0) (Figure 4).

**Figure 4:**
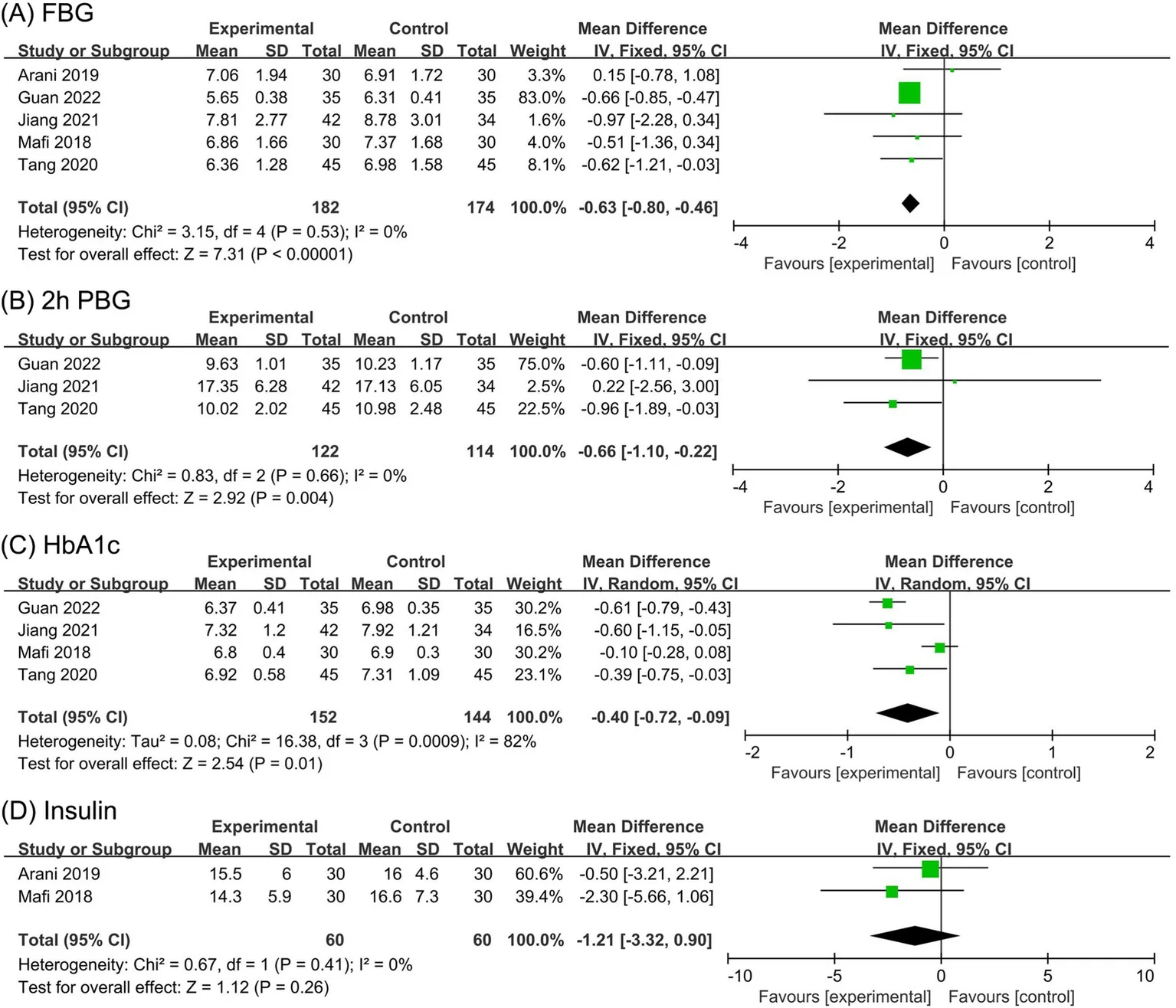
Forest plots of the meta-analysis on glucose metabolism outcomes: **(A)** FBG; **(B)** 2 h PBG; **(C)** HbA1c; **(D)** Insulin. The diamond represents the pooled effect estimate; squares represent individual study estimates with size proportional to study weight. MD, mean difference; CI, confidence interval; FBG, fasting blood glucose; 2 h PBG, 2-h postprandial blood glucose; HbA1c, glycosylated hemoglobin.

#### 3.4.3 Lipid metabolism outcomes

Compared with standard therapy alone, probiotic supplementation was associated with significant reductions in lipid metabolism indicators, including TG (MD −19.17 mg/dL, 95% CI −35.14 to −3.20, *p* = 0.02, *I*^2^ = 0), TC (MD −11.68 mg/dL, 95% CI −20.37 to −2.99, *p* = 0.008, *I*^2^ = 0), and LDL-C (MD −12.72 mg/dL, 95% CI −18.76 to −6.67, *p* < 0.0001, *I*^2^ = 0). However, no statistically significant difference was observed in HDL-C levels between the probiotic and control groups (MD 3.05 mg/dL, 95% CI −0.03 to 6.14, *p* = 0.05, *I*^2^ = 57%) (Figure 5).

**Figure 5:**
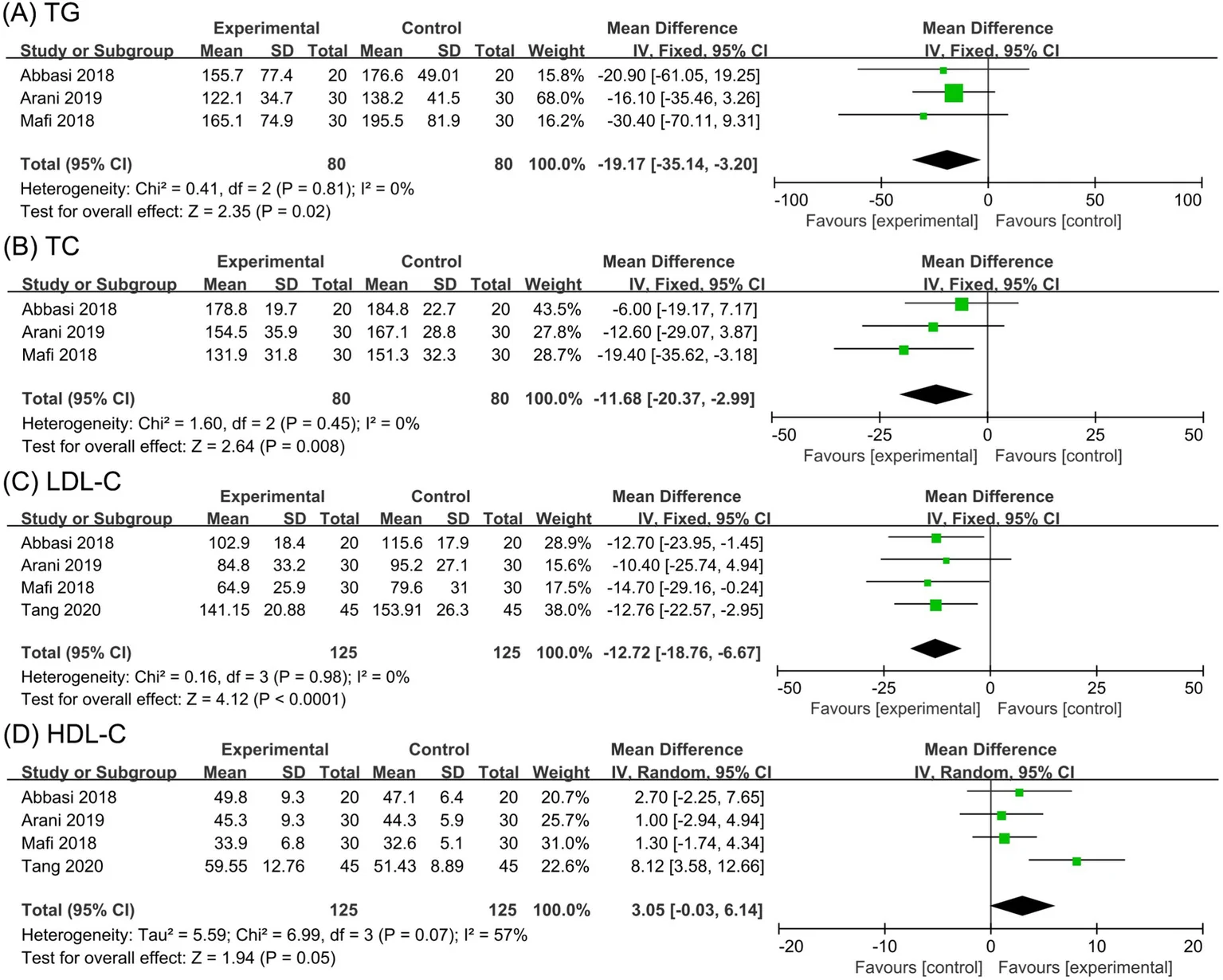
Forest plots of the meta-analysis on lipid metabolism outcomes: **(A)** TG; **(B)** TC; **(C)** LDL-C; **(D)** HDL-C. The diamond represents the pooled effect estimate; squares represent individual study estimates with size proportional to study weight. MD, mean difference; CI, confidence interval; TG, triglycerides; TC, total cholesterol; LDL-C, low-density lipoprotein cholesterol; HDL-C, high-density lipoprotein cholesterol.

#### 3.4.4 Inflammation and oxidative outcomes

Compared with standard therapy alone, probiotic supplementation was associated with significant improvements in inflammatory and oxidative stress biomarkers, including reductions in hs-CRP (MD −1.69 mg/L, 95% CI −2.38 to −1.00, *p* < 0.00001, *I*^2^ = 0) and MDA (MD −0.52 μmol/L, 95% CI −0.91 to −0.13, *p* = 0.008, *I*^2^ = 91%). However, no statistically significant difference was observed in TAC levels between the probiotic and control groups (MD 14.04 mmol/L, 95% CI −7.46 to 35.54, *p* = 0.20, *I*^2^ = 0) (Figure 6).

**Figure 6:**
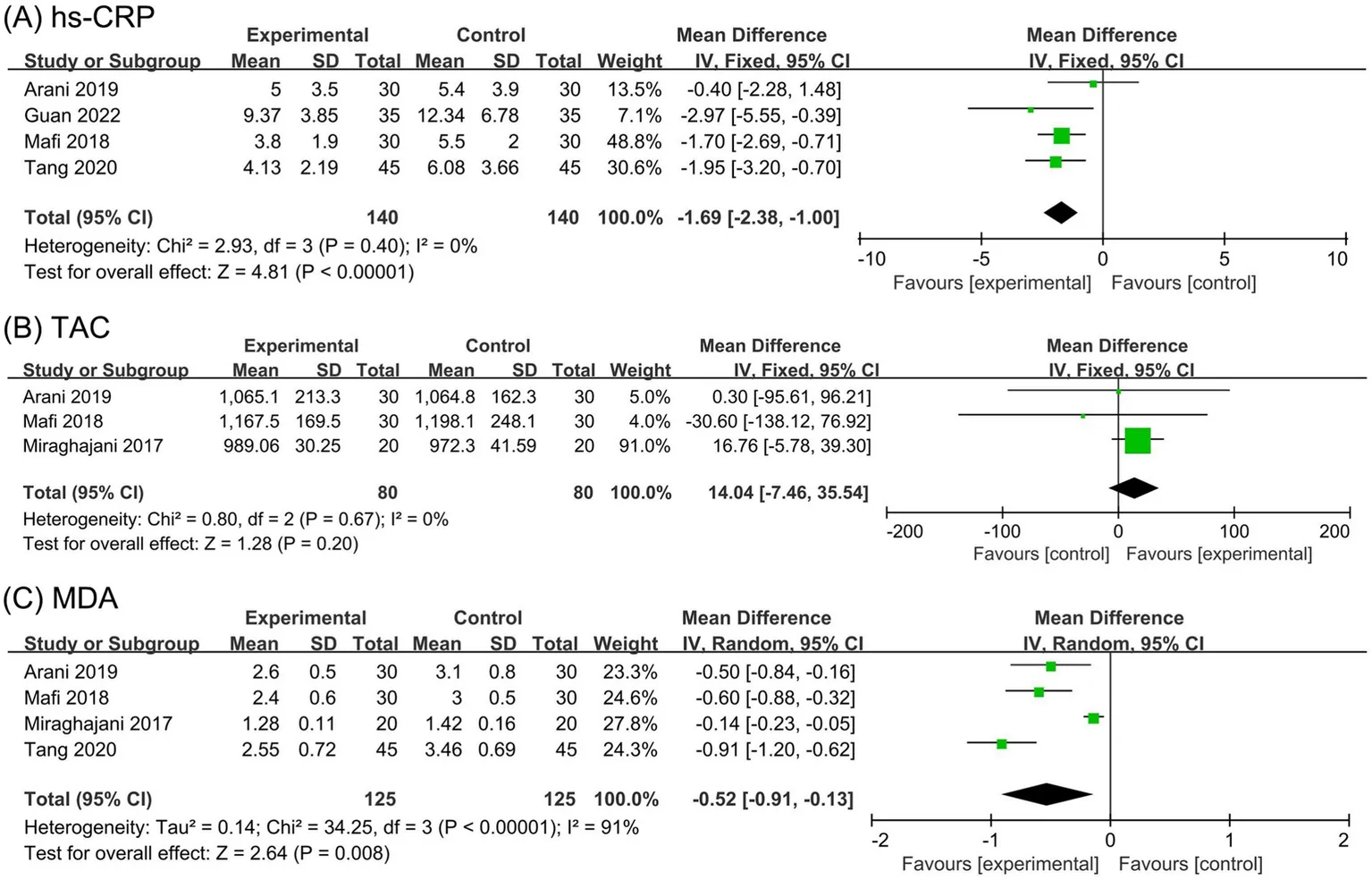
Forest plots of the meta-analysis on inflammation and oxidative outcomes: **(A)** Hs-CRP; **(B)** TAC; **(C)** MDA. The diamond represents the pooled effect estimate; squares represent individual study estimates with size proportional to study weight. MD, mean difference; CI, confidence interval; Hs-CRP, high-sensitivity C-reactive protein; TAC, total antioxidant capacity; MDA, malondialdehyde.

### 3.5 Sensitivity analysis

Substantial heterogeneity was detected in the pooled analyses of eGFR, BUN, HbA1c, HDL-C, and MDA. Accordingly, leave-one-out sensitivity analyses were performed to identify potential sources of heterogeneity and to assess the robustness of the pooled estimates (Table 2).

**Table 2:** Leave-one-out sensitivity analyses of high heterogeneity outcomes.

Outcome	Heterogeneity source	Sensitivity analysis results			Robustness
		*I*^2^/%	MD (95% CI)	*p*-value	
eGFR	None	–	–	–	Not robust
BUN	Lu (2024) (33)	0	−1.58 (−2.80, −0.36)	0.01	Robust
HbA1c	Mafi (2018) (34)	0	−0.57 (−0.72, −0.42)	<0.00001	Robust
HDL-C	Tang (2020) (36)	0	1.48 (−0.69, 3.64)	0.18	Robust
MDA	Miraghajani 2017 (35)	47	−0.68 (−0.92, −0.44)	<0.00001	Robust

MD, mean difference; CI, confidence interval; eGFR, estimated glomerular filtration rate; BUN, blood urea nitrogen; HbA1c, glycosylated hemoglobin; HDL-C, high-density lipoprotein cholesterol; MDA, malondialdehyde.

In the analysis of BUN, the study by Lu et al. (33) was identified as the main contributor to heterogeneity, potentially due to its short intervention duration of only 4 weeks. Removal of this study resulted in a marked reduction in heterogeneity while the statistical significance of the pooled effect was maintained (MD −1.58, 95% CI −2.80 to −0.36, *p* = 0.01, *I*^2^ = 0%), indicating that the observed reduction in BUN was robust.

Regarding HbA1c, heterogeneity was mainly attributable to the study by Mafi et al. (34), possibly reflecting regional differences, as participants were recruited exclusively from Iran. Exclusion of this study substantially reduced heterogeneity without altering the direction or significance of the effect estimate (MD −0.57%, 95% CI −0.72 to −0.42, *p* < 0.00001, *I*^2^ = 0%), supporting the robustness of the HbA1c findings.

For HDL-C, sensitivity analysis identified the study by Tang et al. (36) as the primary source of heterogeneity, which may be related to the fact that the study population was limited to China. After omitting this study, heterogeneity was eliminated while the pooled result remained non-significant (MD 1.48, 95% CI − 0.69 to 3.64, *p* = 0.18, *I*^2^ = 0%), indicating that the HDL-C result was stable.

In the analysis of MDA, heterogeneity was largely explained by the study conducted by Miraghajani et al. (35), potentially due to its relatively short intervention period of 8 weeks. Following exclusion of this study, heterogeneity was substantially reduced while the pooled effect remained statistically significant (MD −0.68, 95% CI −0.92 to −0.44, *p* < 0.00001, *I*^2^ = 47%), suggesting robust results for MDA.

Additionally, leave-one-out sensitivity analyses were used to evaluate the stability of pooled estimates across all included outcomes. The pooled effects of SCR, FBG, HbA1c, insulin, LDL-C, HDL-C, hs-CRP, TAC, and MDA remained stable after exclusion of individual studies, whereas the results for 24-h urine protein, eGFR, BUN, 2 h PBG, TG, and TC showed greater sensitivity to individual studies.

### 3.6 Subgroup analysis

Subgroup analyses were performed to explore how clinical factors such as participant source, average age, probiotic dosage, and treatment duration influence the high heterogeneity observed in outcomes, including eGFR, BUN, HbA1c, HDL-C, and MDA levels, as detailed in Table 3.

**Table 3:** Subgroup analyses of high heterogeneity outcomes.

Outcome	Subject	Subgroup	Number of studies	*I*^2^/%	MD (95% CI)	*P*-value
eGFR	Participant source	China	2	96	4.58 (−7.28, 16.45)	0.45
		Malaysia	1	0	4.18 (−1.01, 9.37)	0.11
		Iran	1	0	12.10 (4.19, 20.01)	0.003
	Average age	≥60 years	3	58	8.76 (4.09, 13.44)	0.0002
		<60 years	1	0	−1.48 (−5.04, 2.08)	0.42
	Probiotic dosage	≥4 × 10^9^ CFU/day	2	63	7.56 (−0.12, 15.23)	0.054
		<4 × 10^9^ CFU/day	2	96	4.58 (−7.28, 16.45)	0.45
	Treatment duration	≥12 weeks	2	68	1.02 (−4.49, 6.53)	0.72
		<12 weeks	2	0	10.87 (7.70, 14.03)	< 0.00001
BUN	Participant source	China	2	94	−3.51 (−7.13, 0.12)	0.06
		Iran	2	0	−1.23 (−4.18, 1.72)	0.41
	Average age	≥60 years	2	83	−3.28 (−7.90, 1.34)	0.16
		<60 years	2	0	−1.71 (−3.00, −0.41)	0.01
	Probiotic dosage	≥4 × 10^9^ CFU/day	3	89	−2.76 (−5.78, 0.26)	0.07
		<4 × 10^9^ CFU/day	1	0	−2.60 (−7.85, 2.65)	0.33
	Treatment duration	≥12 weeks	3	0	−1.58 (−2.80, −0.36)	0.01
		<12 weeks	1	0	−5.35 (−6.63, −4.07)	<0.00001
HbA1c	Participant source	China	3	0	−0.57 (−0.72, −0.42)	<0.00001
		Iran	1	0	−0.10 (−0.28, 0.08)	0.27
	Average age	≥60 years	1	0	−0.61 (−0.79, −0.43)	<0.00001
		<60 years	3	54	−0.29 (−0.57, 0.00)	0.050
	Probiotic dosage	≥4 × 10^9^ CFU/day	3	0	−0.57 (−0.72, −0.42)	<0.00001
		<4 × 10^9^ CFU/day	1	0	−0.10 (−0.28, 0.08)	0.27
HDL-C	Participant source	China	3	64	3.87 (−0.44, 8.18)	0.08
		Iran	1	0	1.30 (−1.74, 4.34)	0.40
		≥60 years	1	0	1.00 (−2.94, 4.94)	0.62
		<60 years	3	67	3.84 (−0.30, 7.99)	0.07
	Probiotic dosage	≥4 × 10^9^ CFU/day	2	0	1.68 (−0.91, 4.28)	0.20
		<4 × 10^9^ CFU/day	2	81	4.47 (−2.51, 11.44)	0.21
	Treatment duration	≥12 weeks	3	71	3.24 (−0.88, 7.35)	0.12
		<12 weeks	1	0	2.70 (−2.25, 7.65)	0.28
MDA	Participant source	China	1	0	−0.91 (−1.20, −0.62)	<0.00001
		Iran	3	84	−0.39 (−0.73, −0.05)	0.02
	Average age	≥60 years	1	0	−0.50 (−0.84, −0.16)	0.004
		<60 years	3	94	−0.54 (−1.03, −0.04)	0.03
	Probiotic dosage	≥4 × 10^9^ CFU/day	2	89	−0.35 (−0.80, 0.10)	0.13
		<4 × 10^9^ CFU/day	2	69	−0.71 (−1.12, −0.31)	0.0005
	Treatment duration	≥12 weeks	3	47	−0.68 (−0.92, −0.44)	<0.00001
		<12 weeks	1	0	−0.14 (−0.23, −0.05)	0.001

MD, mean difference; CI, confidence interval; eGFR, estimated glomerular filtration rat; BUN, blood urea nitrogen; HbA1c, glycosylated hemoglobin; HDL-C, high-density lipoprotein cholesterol; MDA, malondialdehyde.

For BUN, heterogeneity appeared to be associated with treatment duration. Specifically, probiotic supplementation significantly reduced BUN levels in DN patients regardless of treatment duration: ≥12 weeks (MD −1.58, 95% CI −2.80 to −0.36, *p* = 0.01, *I*^2^ = 0) and <12 weeks (MD −5.35, 95% CI −6.63 to −4.07, *p* < 0.00001, *I*^2^ = 0).

The heterogeneity of HbA1c seemed to be related to participant source and probiotic dosage. Probiotics significantly reduced HbA1c in Chinese DN patients (MD −0.57%, 95% CI − 0.72 to −0.42, *p* < 0.00001, *I*^2^ = 0), whereas no significant effect was observed in Iranian patients (MD −0.10%, 95% CI −0.28 to 0.08, *p* = 0.27, *I*^2^ = 0). Similarly, supplementation with ≥4 × 10^9^ CFU/day probiotics decreased HbA1c levels (MD −0.57%, 95% CI −0.72 to −0.42, *p* < 0.00001, *I*^2^ = 0), while <4 × 10^9^ CFU/day showed no significant effect (MD −0.10%, 95% CI − 0.28 to 0.08, *p* = 0.27, *I*^2^ = 0).

For MDA, heterogeneity appeared to be related to treatment duration. Probiotic supplementation reduced MDA levels in DN patients for both ≥12 weeks (MD −0.68, 95% CI − 0.92 to −0.44, *p* < 0.00001, *I*^2^ = 47%) and <12 weeks (MD −0.14, 95% CI − 0.23 to −0.05, *p* = 0.001, *I*^2^ = 0).

In summary, the heterogeneity in BUN and MDA may be attributable to treatment duration, HDL-C heterogeneity may be related to participant source, and HbA1c heterogeneity may be influenced by both participant source and probiotic dosage. However, neither sensitivity nor subgroup analyses identified potential methodological or clinical sources of heterogeneity for eGFR, suggesting that the observed heterogeneity for this outcome may be statistical in nature.

### 3.7 TSA

The TSA was conducted to assess the reliability and validity of the meta-analysis results, aiming to minimize the risk of false-positive findings. The cumulative Z-curves for SCR, FBG, 2 h PBG, HbA1c, TC, LDL-C, hs-CRP, and MDA crossed the predefined monitoring boundaries, suggesting that these outcomes may have reached the required information size. However, these findings should be interpreted cautiously. Specifically, for 2 h PBG and TC, the TSA results were inconsistent with the sensitivity analyses, which indicated that the pooled effects were unstable and heavily influenced by individual studies. This discrepancy implies that crossing the TSA monitoring boundary does not necessarily guarantee clinical reliability, particularly when effect sizes are small, heterogeneity is high, or results are driven by single influential trials. In contrast, the Z-curves for BUN and TG did not cross the monitoring boundaries, indicating that further high-quality studies are required to confirm these findings (Figure 7).

**Figure 7:**
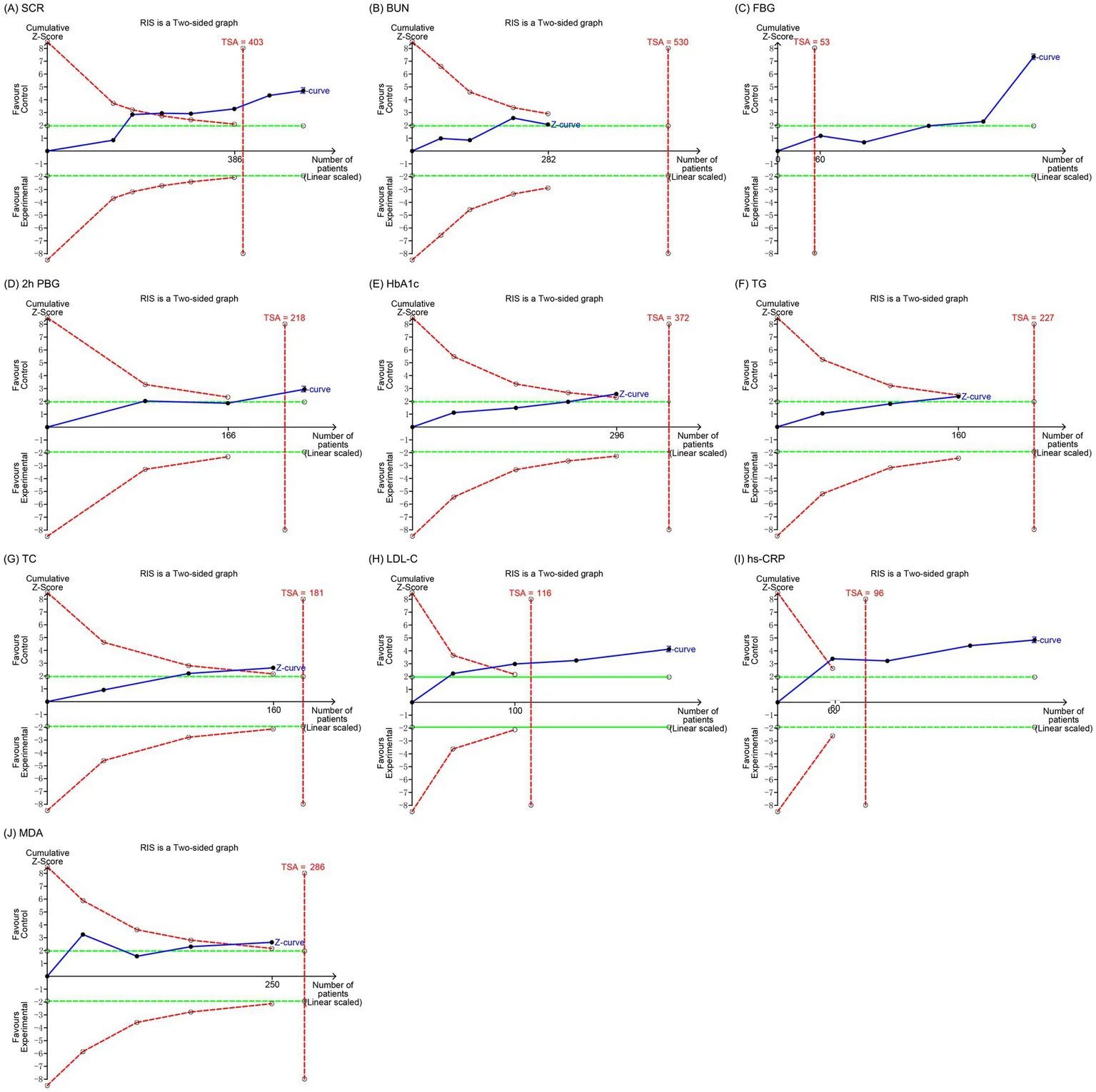
Trial sequential analyses of positive outcomes: **(A)** SCR; **(B)** BUN; **(C)** FBG; **(D)** 2 h PBG; **(E)** HbA1c; **(F)** TG; **(G)** TC; **(H)** LDL-C; **(I)** hs-CRP; **(J)** MDA. The red cumulative Z-curve represents the sequential meta-analysis; The horizontal green lines denote the conventional significance boundary; The red inward-sloping lines represent the TSA-adjusted monitoring boundaries; The blue vertical line indicates the required information size. When the cumulative Z-curve crosses the TSA monitoring boundary, the evidence is considered conclusive despite sparse data and repetitive testing. SCR, serum creatinine; BUN, blood urea nitrogen; FBG, fasting blood glucose; 2 h PBG, 2-h postprandial blood glucose; HbA1c, glycosylated hemoglobin; TG, triglycerides; TC, total cholesterol; LDL-C, low-density lipoprotein cholesterol; hs-CRP, high-sensitivity C-reactive protein; MDA, malondialdehyde.

### 3.8 Publication bias

Visual inspection of funnel plots suggested potential asymmetry for SCR, eGFR, BUN, 2 h PBG, TG, TAC, and MDA, indicating possible small-study effects or publication bias. In contrast, funnel plots for FBG, HbA1c, TC, LDL-C, HDL-C, and hs-CRP showed relatively symmetrical distributions, suggesting no obvious evidence of asymmetry (Figure 8).

**Figure 8:**
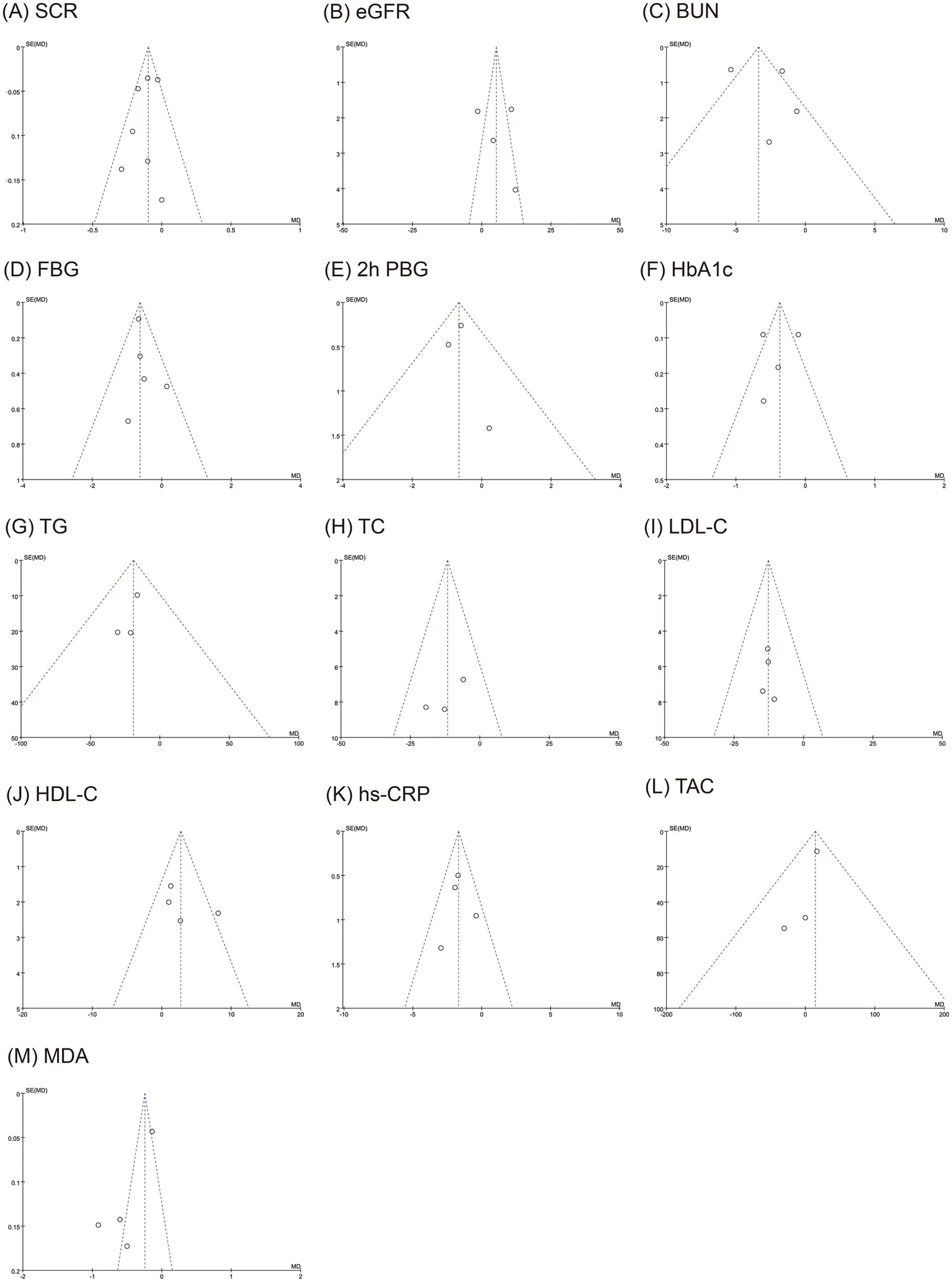
Funnel plots of publication bias: **(A)** SCR; **(B)** eGFR; **(C)** BUN; **(D)** FBG; **(E)** 2 h PBG; **(F)** HbA1c; **(G)** TG; **(H)** TC; **(I)** LDL-C; **(J)** HDL-C; **(K)** hs-CRP; **(L)** TAC; **(M)** MDA. Each dot represents an individual study included in the meta-analysis. The horizontal axis indicates the effect estimate, and the vertical axis represents the standard error of the effect estimate. SCR, serum creatinine; eGFR, estimated glomerular filtration rate; BUN, blood urea nitrogen; FBG, fasting blood glucose; 2 h PBG, 2-h postprandial blood glucose; HbA1c, glycosylated hemoglobin; TG, triglycerides; TC, total cholesterol; LDL-C, low-density lipoprotein cholesterol; HDL-C, high-density lipoprotein cholesterol; hs-CRP, high-sensitivity C-reactive protein; TAC, total antioxidant capacity; MDA, malondialdehyde.

However, Egger’s tests did not identify statistically significant publication bias for SCR (*p* = 0.384), eGFR (*p* = 0.754), BUN (*p* = 0.672), FBG (*p* = 0.464), 2 h PBG (*p* = 0.819), HbA1c (*p* = 0.809), TG (*p* = 0.352), TC (*p* = 0.340), LDL-C (*p* = 0.858), HDL-C (*p* = 0.424), hs-CRP (*p* = 0.961), TAC (*p* = 0.263), or MDA (*p* = 0.083) (Figure 9). Therefore, although some funnel plots showed visual asymmetry, the lack of statistical significance in Egger’s tests suggests that substantial publication bias was not evident for the evaluated outcomes. These analyses were not performed for 24 h UP and insulin because fewer than three studies were available.

**Figure 9:**
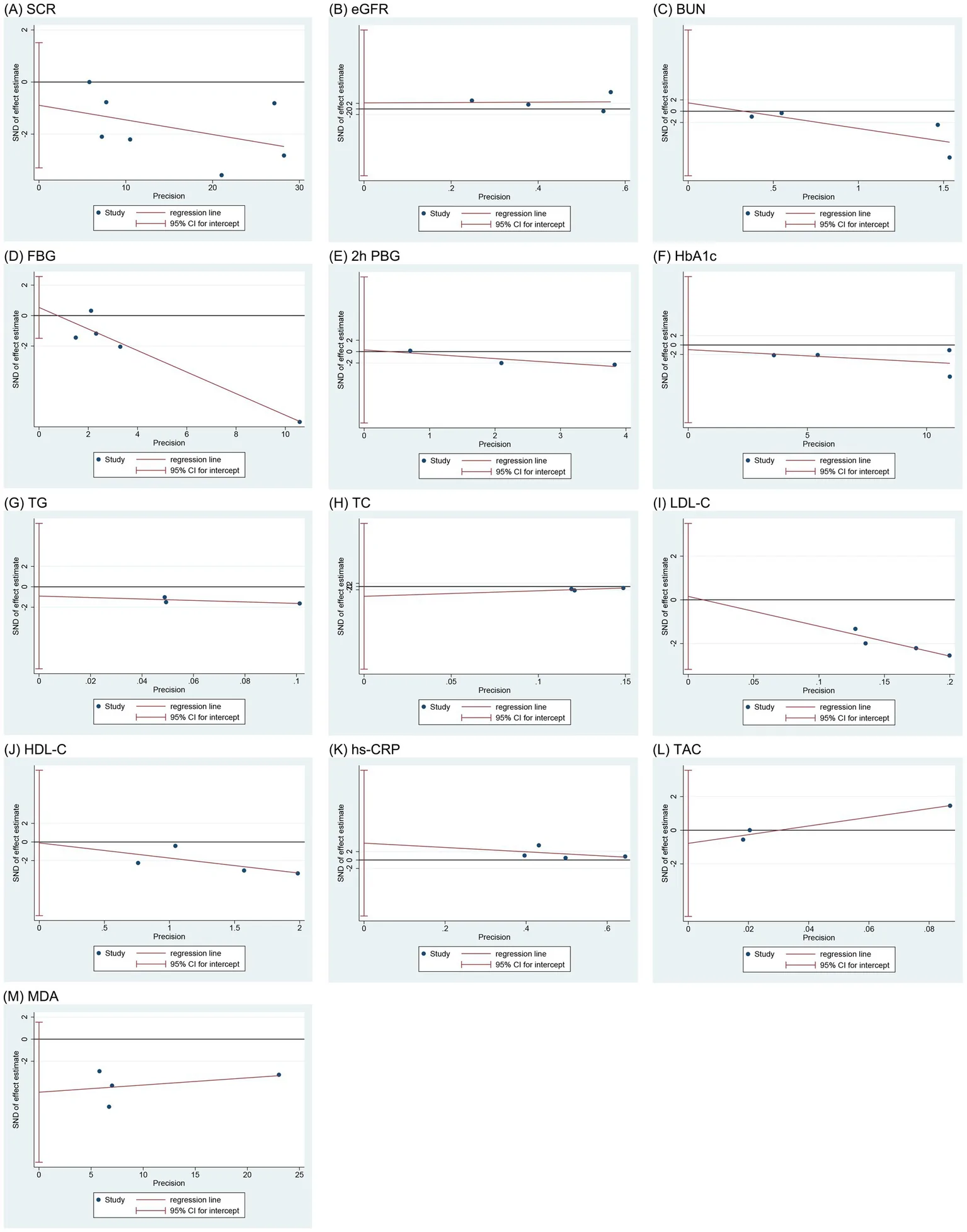
Egger’s test of publication bias: **(A)** SCR; **(B)** eGFR; **(C)** BUN; **(D)** FBG; **(E)** 2 h PBG; **(F)** HbA1c; **(G)** TG; **(H)** TC; **(I)** LDL-C; **(J)** HDL-C; **(K)** hs-CRP; **(L)** TAC; **(M)** MDA. Each funnel plot displays the standardized effect estimate (x-axis) against its precision (y-axis, inverse of standard error). The solid diagonal line represents the regression line from Egger’s test; asymmetrical scatter around this line suggests potential publication bias. SCR, serum creatinine; eGFR, estimated glomerular filtration rate; BUN, blood urea nitrogen; FBG, fasting blood glucose; 2 h PBG, 2-h postprandial blood glucose; HbA1c, glycosylated hemoglobin; TG, triglycerides; TC, total cholesterol; LDL-C, low-density lipoprotein cholesterol; HDL-C, high-density lipoprotein cholesterol; hs-CRP, high-sensitivity C-reactive protein; TAC, total antioxidant capacity; MDA, malondialdehyde.

### 3.9 Certainty of evidence

According to the GRADE approach, the certainty of evidence was rated as moderate for SCR, FBG, LDL-C, and hs-CRP; low for 2 h PBG, TG, TC, TAC, and MDA; and very low for 24 h UP, eGFR, BUN, HbA1c, insulin, and HDL-C (Table 4). The overall strength of recommendation based on the available evidence was considered weak.

**Table 4:** Certainty of evidence.

Outcome	Risk of bias	Inconsistency	Indirectness	Imprecision	Publication bias	MD (95% CI)	Certainty of evidence
SCR	Serious	None	None	None	None	−0.10 (−0.14, −0.06)	Moderate
24 h UP	Serious	None	None	Serious	Suspected	−0.24 (−0.48, 0.01)	Very Low
eGFR	Serious	Serious	None	Serious	None	6.06 (−0.72, 12.83)	Very Low
BUN	Serious	Serious	None	Serious	None	−2.76 (−5.40, −0.11)	Very Low
FBG	Serious	None	None	None	None	−0.63 (−0.80, −0.46)	Moderate
2 h PBG	Serious	None	None	Serious	None	−0.66 (−1.10, −0.22)	Low
HbA1c	Serious	Serious	None	Serious	None	−0.40 (−0.72, −0.09)	Very Low
Insulin	Serious	None	None	Serious	Suspected	−1.21 (−3.32, 0.90)	Very Low
TG	Serious	None	None	Serious	None	−19.17 (−35.14, −3.20)	Low
TC	Serious	None	None	Serious	None	−11.68 (−20.37, −2.99)	Low
LDL-C	Serious	None	None	None	None	−12.72 (−18.76, −6.67)	Moderate
HDL-C	Serious	Serious	None	Serious	None	3.05 (−0.03, 6.14)	Very Low
hs-CRP	Serious	None	None	None	None	−1.69 (−2.38, −1.00)	Moderate
TAC	Serious	None	None	Serious	None	14.04 (−7.46, 35.54)	Low
MDA	Serious	Serious	None	None	None	−0.52 (−0.91, −0.13)	Low

SCR, serum creatinine; 24 h UP, 24-h urine protein; eGFR, estimated glomerular filtration rate; BUN, blood urea nitrogen; FBG, fasting blood glucose; 2 h PBG, 2-h postprandial blood glucose; HbA1c, glycosylated hemoglobin; TG, triglycerides; TC, total cholesterol; LDL-C, low-density lipoprotein cholesterol; HDL-C, high-density lipoprotein cholesterol; hs-CRP, high-sensitivity C-reactive protein; TAC, total antioxidant capacity; MDA, malondialdehyde.

## 4 Discussion

### 4.1 Research significance and findings

In recent years, increasing attention has been paid to the role of probiotics as an adjunctive therapy in DN. Although previous meta-analyses have provided preliminary evidence supporting the use of probiotics in patients with DN, concerns have been raised regarding the robustness and clinical generalizability of their findings due to potential confounding factors, the lack of TSA adjustment, and inconsistent results across studies. Therefore, in the present meta-analysis, we excluded studies involving uremic populations and incorporated TSA to adjust the pooled estimates. Our findings demonstrated that probiotics significantly reduced SCR, FBG, LDL-C, hs-CRP, and MDA levels in patients with DN, while the effects on other outcomes remain to be further evaluated and validated.

### 4.2 Effect of probiotics on renal function

The SCR, BUN, 24 h UP, and eGFR are commonly used clinical indicators for assessing renal function. Our meta-analysis showed that, compared with the control group, patients receiving probiotics had significantly reduced levels of SCR and BUN, whereas no significant differences were observed in 24 h UP and eGFR. Sensitivity analyses further indicated that the reductions in SCR and BUN were robust. Previous studies have reported similar findings. Specifically, the meta-analysis by Dai et al. (22) demonstrated that probiotics significantly reduced SCR (MD −0.17, 95% CI −0.29 to −0.05) and BUN (MD −1.36, 95% CI −2.20 to −0.52), while Liu et al. (19) also reported significant reductions in SCR (MD −0.09, 95% CI −0.14 to −0.04) and BUN (MD −1.58, 95% CI −2.80 to −0.36). Both studies consistently found no significant effects on 24 h UP and eGFR, which aligns with our results (19, 22). However, Tarrahi et al. (20) reported no significant effect of probiotics on BUN (MD −1.49, 95% CI −4.31 to 1.33, *p* = 0.301). This discrepancy may be attributed to the limited sample size, as only three studies were included in that analysis. Notably, TSA indicated that the evidence for SCR reduction was conclusive, whereas the cumulative sample size for BUN did not reach the required threshold. This suggests that the previously reported benefit of BUN is inconclusive and should be interpreted with caution. Overall, probiotics reduce SCR levels in patients with DN, while their effect on BUN requires further investigation.

Creatinine is primarily generated from the breakdown of creatine during energy metabolism in muscle, whereas urea is produced in the liver through the combination of ammonia derived from amino acid deamination and carbon dioxide (37). Both are nitrogenous end-products of metabolism that enter the bloodstream and are mainly excreted through glomerular filtration (37), with a small proportion diffusing into the gut where they are metabolized and recycled by the gut microbiota (38, 39). Microorganisms such as *Bacillus*, *Clostridium*, *Corynebacterium*, *Cryptococcus neoformans*, and *Alcaligenes* possess enzymatic activities including creatinase, creatine amidinohydrolase, and creatinine deaminase, enabling the conversion of creatinine into 1-methylhydantoin, ammonia, urea, and other metabolites (38). Meanwhile, bacteria such as *Bacteroides cellulosilyticus* WH2, *Coprococcus comes*, *Roseburia intestinalis*, *Streptococcus infantarius*, and *S. thermophilus* produce urease, allowing them to hydrolyze and utilize approximately 15–30% of urea (39–41). However, in patients with DN, gut microbiota dysbiosis reduces the capacity for creatinine and urea degradation, leading to their accumulation in the body. In contrast, probiotic supplementation has been shown to improve the composition and diversity of gut microbiota in diabetic animal models, reduce the abundance of harmful bacteria, and promote the metabolism of creatinine and urea (42).

### 4.3 Effect of probiotics on glycolipid metabolism

FBG, 2 h PBG, HbA1c, and insulin are key indicators for evaluating glucose metabolism. Our meta-analysis showed that, compared with the control group, patients receiving probiotics had significantly reduced levels of FBG, 2 h PBG, and HbA1c, whereas no significant difference was observed in insulin levels. Sensitivity analyses further confirmed that the results for FBG, HbA1c, and insulin were robust, while those for 2 h PBG were not stable. Previous studies have reported similar findings. Meta-analyses by Tarrahi et al. (20), Dai et al. (22), and Liu et al. (19) consistently demonstrated that probiotics significantly reduced FBG, and Dai et al. (22) also reported a significant reduction in HbA1c (MD −0.12%, 95% CI 0.00 to 0.03), in line with our findings. However, Tarrahi et al. (20) and Liu et al. (19) reported negative results for 2 h PBG and HbA1c. These discrepancies may be attributed to differences in participant characteristics and sample sizes. Specifically, Tarrahi et al. (20) included uremic patients undergoing hemodialysis, who typically follow specific dietary patterns characterized by low protein, low phosphorus, low potassium, and low sodium intake. Such dietary patterns may positively influence glucose metabolism, thereby attenuating the observable effects of probiotics. In addition, Liu et al. (19) included only two or three studies for 2 h PBG and HbA1c and omitted data from Guan (31), which may have introduced selection bias and increased the risk of type II error. Although TSA indicated that the cumulative evidence for FBG, 2 h PBG, and HbA1c reached the required information size, the lack of robustness in sensitivity analyses undermines the reliability of the findings for 2 h PBG. Overall, probiotics significantly reduce FBG levels in patients with DN, whereas their effects on 2 h PBG and HbA1c require further validation.

TG, TC, LDL-C, and HDL-C are commonly used clinical indicators for assessing lipid metabolism. Our meta-analysis demonstrated that probiotics significantly reduced TG, TC, and LDL-C levels, while no significant effect was observed on HDL-C. Sensitivity analyses supported the robustness of LDL-C and HDL-C, but not TG and TC. Previous studies have reported comparable findings. Liu et al. (19) showed that probiotics significantly reduced TG (MD −19.17, 95% CI −35.14 to −3.20), TC (MD −11.68, 95% CI −20.37 to −2.99), and LDL-C (MD −12.72, 95% CI −18.76 to −6.67), with no significant effect on HDL-C, which is consistent with our results. In contrast, Dai et al. (22) reported a significant increase in HDL-C (MD 2.72, 95% CI 0.47 to 4.97) but no reduction in TG, indicating conflicting findings. Notably, the reported benefit in HDL-C by Dai et al. (22) was observed after including the study by Soleimani et al. (43), which was limited to uremic patients undergoing dialysis. This may have introduced additional confounding factors and led to an overestimation of the effect of probiotics on HDL-C. TSA showed that the cumulative sample size for LDL-C reached the required threshold, whereas TG did not. Therefore, unlike Liu et al. (19), the present study takes a conservative stance on TC and TG, as these outcomes did not withstand sensitivity analyses and TSA. In summary, probiotics significantly reduce LDL-C levels in patients with DN, whereas their effects on TC and TG are not robust.

Previous evidence suggests that probiotics can regulate glycolipid metabolism through multiple mechanisms (44–46). First, certain probiotic strains, such as *Hafnia alvei HA4597*, exhibit strong glucose-metabolizing capacity and may act as a barrier in glucose regulation (47). Second, probiotics can enhance the expression of intestinal tight junction proteins and repair impaired intestinal barrier function, thereby reducing the translocation of endotoxins such as lipopolysaccharide (LPS) and improving systemic inflammation (48). Third, probiotics ferment dietary fiber to produce short-chain fatty acids, including acetate, propionate, and butyrate, which can promote the release of glucagon-like peptide-1 and peptide YY, suppress appetite, reduce hepatic glucose production, enhance insulin secretion, and improve insulin sensitivity (49). Fourth, probiotics such as *Akkermansia muciniphila*, *Bacteroides* spp., *Clostridium* spp., *Christensenella minuta*, *Eubacterium* spp., and *Faecalibacterium prausnitzii* can regulate cholesterol absorption and metabolism through their metabolites, inhibit hepatic cholesterol synthesis, and promote cholesterol conversion and incorporation into cell membranes (44). Collectively, these mechanisms confer probiotics with the potential to modulate glycolipid metabolism.

### 4.4 Effect of probiotics on inflammation and oxidative stress

The present meta-analysis demonstrated that probiotic supplementation exerts significant anti-inflammatory and antioxidant effects in patients with DN. Compared with the control group, probiotics significantly reduced hs-CRP and MDA levels, although no significant difference was observed in TAC. Sensitivity analyses indicated that the reductions in hs-CRP and MDA were robust. TSA further confirmed that the cumulative evidence for hs-CRP and MDA reached the required information size, supporting the reliability of these findings. In previous meta-analyses, AbdelQadir et al. (23) reported that probiotics significantly reduced hs-CRP (MD −1.55, 95% CI −2.19 to −0.92) and MDA (MD −0.77, 95% CI −0.96 to −0.58), while significantly increasing TAC (MD 62.29, 95% CI 18.34 to 106.24). Similarly, Bohlouli et al. (21) found significant reductions in hs-CRP (MD −1.53, 95% CI −2.38 to −0.69) and MDA (MD −0.62, 95% CI −1.18 to −0.06), along with a significant increase in TAC (MD 26.54, 95% CI 6.23 to 46.85). Dai et al. (22) also reported significant reductions in hs-CRP (MD −1.55, 95% CI −2.19 to −0.91) and MDA (MD −0.66, 95% CI −1.16 to −0.16), as well as an increase in TAC (MD 0.64, 95% CI 0.41 to 0.87). However, the reported benefit in TAC may have been overestimated, as these studies all included uremic patients from the study by Soleimani et al. (43), which may have confounded the true effects of probiotics. In contrast, the meta-analysis by Liu et al. (19), which excluded the study by Soleimani et al. (43), reported no significant effect on TAC, supporting this interpretation. Overall, probiotics effectively improve hs-CRP and MDA levels in patients with DN, whereas their effect on TAC requires further validation.

Inflammation and oxidative stress are key pathological mechanisms underlying DN. Chronic hyperglycemia disrupts gut microbiota homeostasis and impairs intestinal barrier function, leading to the translocation of endotoxins such as LPS, which activates Toll-like receptor 4 (TLR4) and downstream nuclear factor-κB (NF-κB) signaling pathways. This cascade promotes the release of pro-inflammatory mediators, including TNF-α, IL-1, and adhesion molecules, which in turn recruit inflammatory cells such as macrophages and monocytes into renal tissue, ultimately inducing renal inflammation and oxidative stress damage (48, 50, 51). Meanwhile, hyperglycemia accelerates the accumulation of advanced glycation end products and sorbitol, which stimulate reactive oxygen species (ROS) production and consume nicotinamide adenine dinucleotide phosphate (NADPH) oxidase, thereby contributing to renal injury and fibrosis (52). Probiotics exert renoprotective effects by modulating inflammation and oxidative stress through multiple pathways. First, probiotics restore gut microbiota balance and enhance intestinal epithelial barrier integrity, thereby reducing endotoxemia and blocking upstream activation of pro-inflammatory signaling (53). Second, probiotics suppress the transcription of pro-inflammatory genes through epigenetic mechanisms, including the regulation of histone deacetylation and microRNA expression (54). In addition, probiotics activate nuclear factor erythroid 2–related factor 2 (Nrf2), inducing the expression of downstream antioxidant genes and enhancing intracellular ROS scavenging capacity (55). Moreover, probiotic strains such as *Lactobacilli* and *Bifidobacteria* can directly inhibit oxidase activity and increase superoxide dismutase activity, thereby reducing ROS generation and promoting their clearance (56). Collectively, by restoring the intestinal barrier, regulating inflammatory gene expression, and activating cellular antioxidant defense systems, probiotics interrupt the vicious cycle of inflammation and oxidative stress in DN.

### 4.5 Clinical discovery and inspiration

Compared with previous meta-analyses, the present study provides a more conservative and methodologically rigorous evaluation of probiotic supplementation in DN. By excluding studies involving uremic populations and incorporating TSA, we were able to reappraise the robustness of previously reported benefits. Notably, several outcomes that had been considered beneficial in earlier meta-analyses, such as BUN, TG, and TC, were not supported in our analysis due to lack of robustness or failure to reach the required information size. These findings suggest that earlier conclusions may have overestimated the clinical efficacy of probiotics in DN, highlighting the importance of rigorous methodological control in evidence synthesis. More importantly, the clinical relevance of the observed benefits warrants careful interpretation. Although probiotics were associated with statistically significant reductions in SCR, FBG, and LDL-C, their effects (−0.10 mg/dL, −0.63 mmol/L, and −12.72 mg/dL, respectively) did not reach the corresponding minimal clinically important difference (MCID) thresholds (0.30 mg/dL, 1.6 mmol/L, and 18 mg/dL, respectively) (57, 58). This discrepancy indicates that the improvements in renal function and glycolipid metabolism may be quantitatively modest and potentially insufficient to translate into meaningful clinical benefits.

Taken together, these findings suggest that the primary therapeutic value of probiotics in DN may not lie in directly improving glycemic control or lipid metabolism, but rather in modulating systemic inflammation and oxidative stress. This interpretation is supported by the consistent and robust reductions observed in hs-CRP and MDA, both of which are key mediators in the progression of DN. Therefore, probiotics may serve as an adjunctive strategy targeting the inflammatory–oxidative axis, rather than as a primary metabolic regulator.

From a practical perspective, the included studies employed heterogeneous probiotic regimens with respect to strain composition, daily dosage, and treatment duration. Most trials used multispecies formulations containing Lactobacillus and Bifidobacterium species rather than single-strain preparations. Our subgroup analyses suggested that higher probiotic doses (≥4 × 10^9^ CFU/day) were associated with greater reductions in HbA1c, while interventions lasting at least 12 weeks appeared to produce more favorable effects on BUN and MDA than shorter treatment courses. However, because of the limited number of studies and considerable heterogeneity in probiotic formulations, no specific strain, optimal dosage, or treatment duration can currently be recommended for routine clinical practice. Future large-scale, well-designed multicenter RCTs are required to determine the optimal probiotic regimen and to establish whether these biological effects can ultimately improve long-term renal outcomes.

### 4.6 Limitations and prospects

Several limitations of this study should be acknowledged. First, although all included studies were RCTs, the overall risk of bias in certain domains remains a concern. In particular, the lack of placebo control in several trials may have introduced performance bias. Second, the robustness of some outcomes was insufficient. Although statistically significant reductions were observed in 2 h PBG, HbA1c, TG, and TC, these findings were not consistently supported by sensitivity analyses. This suggests that the observed effects may be driven by individual studies or small sample sizes, and therefore should be interpreted with caution. Third, the conclusiveness of certain findings remains limited despite statistical significance. TSA indicated that the cumulative evidence for BUN and TG did not reach the required information size, suggesting a risk of random error and false-positive findings. Finally, the external generalizability of our findings is constrained by the demographic characteristics of the included populations. Most of the studies were conducted in Asian populations, with a notable lack of data from European and African populations. Given the potential influence of genetic background, dietary patterns, and gut microbiota composition on probiotic efficacy, this finding may only apply to Asians.

Future large-scale, multicenter RCTs with rigorous placebo-controlled designs are warranted to validate these findings. Particular attention should be given to improving methodological quality, ensuring adequate sample sizes for key outcomes, and including more ethnically diverse populations. Moreover, future research should further elucidate the mechanistic pathways underlying the anti-inflammatory and antioxidant effects of probiotics, and determine whether these biological improvements can ultimately translate into meaningful long-term renal outcomes in patients with DN.

## 5 Conclusion

The current evidence suggests that probiotic supplementation may be associated with modest improvements in inflammatory and oxidative stress biomarkers in patients with DN. However, its effects on glycolipid metabolism and renal function appear to be limited, and the observed improvements in surrogate biomarkers cannot yet be assumed to translate into meaningful long-term clinical benefits. Moreover, the heterogeneity of probiotic regimens, together with the limited sample sizes and geographic concentration of the available evidence, precludes firm recommendations regarding the optimal probiotic strategy. Therefore, well-designed, large-scale multicenter RCTs using standardized probiotic formulations and involving more diverse populations are required to confirm these findings and to determine their clinical relevance.

## Data Availability

The datasets presented in this study can be found in online repositories. The names of the repository/repositories and accession number(s) can be found in the article/supplementary material.
